# A Motion Artifact Correction Procedure for fNIRS Signals Based on Wavelet Transform and Infrared Thermography Video Tracking

**DOI:** 10.3390/s21155117

**Published:** 2021-07-28

**Authors:** David Perpetuini, Daniela Cardone, Chiara Filippini, Antonio Maria Chiarelli, Arcangelo Merla

**Affiliations:** Department of Neuroscience, Imaging and Clinical Sciences, Institute for Advanced Biomedical Technologies, University G. D’Annunzio of Chieti-Pescara, Via Luigi Polacchi 13, 66100 Chieti, Italy; david.perpetuini@unich.it (D.P.); d.cardone@unich.it (D.C.); chiara.filippini@unich.it (C.F.); antonio.chiarelli@unich.it (A.M.C.)

**Keywords:** motion artefacts, functional near infrared spectroscopy (fNIRS), infrared imaging, infrared thermography, wavelet transform, wavelet coherence, tracking algorithms

## Abstract

Functional near infrared spectroscopy (fNIRS) is a neuroimaging technique that allows to monitor the functional hemoglobin oscillations related to cortical activity. One of the main issues related to fNIRS applications is the motion artefact removal, since a corrupted physiological signal is not correctly indicative of the underlying biological process. A novel procedure for motion artifact correction for fNIRS signals based on wavelet transform and video tracking developed for infrared thermography (IRT) is presented. In detail, fNIRS and IRT were concurrently recorded and the optodes’ movement was estimated employing a video tracking procedure developed for IRT recordings. The wavelet transform of the fNIRS signal and of the optodes’ movement, together with their wavelet coherence, were computed. Then, the inverse wavelet transform was evaluated for the fNIRS signal excluding the frequency content corresponding to the optdes’ movement and to the coherence in the epochs where they were higher with respect to an established threshold. The method was tested using simulated functional hemodynamic responses added to real resting-state fNIRS recordings corrupted by movement artifacts. The results demonstrated the effectiveness of the procedure in eliminating noise, producing results with higher signal to noise ratio with respect to another validated method.

## 1. Introduction

fNIRS is a non-invasive optical methodology able to measure cortical oscillation of oxygenated (O_2_Hb) and deoxygenated (HHb) hemoglobin related to neuronal activity through the blood oxygen level dependent (BOLD) effect [1,2,3]. This technique is portable, relatively cheap, lightweight and quite resilient to motion artifacts with a mechanical structure resembling electroencephalography (EEG) [4,5], thus being suitable for ecological measurements, such as the clinical practice and outdoor applications [6,7,8,9].

However, the subjects’ head movement could corrupt the signal because of the decoupling between the optodes and the scalp, producing an abrupt modification of the light intensity. Improving the optodes–scalp coupling could reduce the motion artefacts entity, but getting rid of these artifacts is quite a challenging issue.

Motion artifacts usually produce high-frequency noise overlapped to the functional hemodynamic signal, but, when the optodes–scalp coupling is definitely compromised, they could also provoke a lasting shift. Hence, movement artifacts may generate both high- and low-frequency components that cannot be easily removed by frequency filtering. Notably, motion artifacts can influence the statistical results, causing a non-reliable identification of functional cortical activity (e.g., general linear model, GLM [10]). For this reason, several procedures for motion artefacts removal have been developed to be applied before statistical inferences [11,12,13].

Two main typologies of motion artifacts correction algorithm have been proposed so far. The first category comprises algorithms that identify large sources of variance in the recording, which are identified as artifacts and then subtracted. These approaches are generally based on principal component analysis (PCA) [14,15], splines [16] or wavelet filtering [17].

The second typology requires the use of an added input signal sensitive to motion artifacts, but not to the functional hemodynamic response, such as an accelerometer [18,19,20,21], a multisensory integrated inertia measurement unit (IMU) [22,23] or an fNIRS channel not sensitive to brain activity [24,25,26]. In order to decompose the data into artifacts and signal, correlation methods and/or adaptive filtering are then generally applied. These approaches exhibit some limitations. A first issue is related to the assumption that the movement effects on the measurement channels is monotonically related to the reference signal used to monitor the movements. However, some movements may corrupt only few channels. Moreover, this approach may not predict the occurrence of permanent shifts in light intensity after a movement.

In order to overcome some of the cited limitation of these algorithms, it could be possible to use as reference signal the movement of each optode, evaluated in a contactless manner, to ensure the absence of interference with the experimental design, to preserve the lightweight and not to increase the encumbrance over the cap. To this aim, a video tracking procedure could be highly suited. Video tracking algorithms based on visible cameras [27] could be employed for this purpose. However, visible cameras do not properly work with poor environmental light, hence alternative methods should be investigated. Infrared thermography (IRT) is a contactless technique able to measure the superficial temperature of an object [28]. IRT was demonstrated to be able to detect the physiological contamination of the fNIRS signal [29], hence providing a tool for the correction of the physiological noise, which is another relevant issue for the fNIRS measurements [30,31]. Thus, a video tracking applied to IRT recordings could allow to improve the quality of the fNIRS data, removing both physiological and motion artifacts.

Of note, tracking algorithms for IRT videos are usually developed for co-registered and synchronous IRT-RGB optics. In fact, the visible video is used to track facial landmarks, and, through a geometrical transformation, these anatomical points are projected onto the IRT frames [32,33,34,35]. Conversely, the literature about tracking algorithms applied on thermal videos is very sparse. The few publications on this theme are about tracking on thermal videos registered on the corresponding visible video, i.e., the tracking is computed on the visible frames and then brought back on the thermal video [36,37,38,39]. Concerning tracking algorithms based on the only IR spectrum videos, in 2003, Eveland et al. [40] developed a method based on a first skin segmentation for detection of faces and then applied the CONDENSATION algorithm for tracking the head regions over time.

In 2007, Dowdall et al. [41] proposed a method that uses a network of independent particle filter trackers whose interactions were modeled using coalitional game theory. Memarian et al. [42], in 2009, realized an IR tracking on the mouth region, based on optical flow, specifically on the Horn–Schunch method. In 2013, Zhou et al. [43] developed a particle filter tracker driven by a probabilistic template function with both spatial and temporal smoothing components, capable of adapting to abrupt positional and physiological changes.

In this paper, an innovative procedure for motion artifacts correction of fNIRS signal is presented. In detail, the procedure is based on the evaluation of the continuous wavelet transform (CWT) of the signal and of the optodes’ movement and on the wavelet coherence (WCOH) between the fNIRS signal and the optodes’ movement. The movement of the fNIRS optodes was detected through a video tracking for IRT recordings. The algorithm performance was tested by employing synthesized hemodynamic response functions (HRF) convolved with a boxcar simulating a functional signal evoked by a block paradigm. The simulated functional signal was added to fNIRS recordings collected from human subjects obtained during resting-state with a controlled head’s movement.

## 2. Materials and Methods

### 2.1. Participants

Sixteen participants were recruited to validate the motion artifacts correction algorithm (mean age ± SD: 25.5 ± 8.5 years; 9 males/7 females). The study was approved by the Research Ethics Board of the University of Chieti-Pescara, and it was conducted according to the principles described in the Declaration of Helsinki. Before the experiment, each participant signed the informed consent form and could withdraw from it at any time. Before the measurement session, each subject was left in the experimental room for 20 min to allow the baseline skin temperature to stabilize. The recording room was set at standardized temperature (23 °C) and humidity (50–60%) by a thermostat.

### 2.2. IRT Instrumentation

The digital thermal infrared camera FLIR SC660 (FLIR, Wilsonville, OR, USA) (640 × 480 bolometer FPA, sensitivity/noise equivalent temperature difference: <30 mK @ 30 °C, field of view: 24° × 18°) was used to track the detectors’ movement. The camera was placed 60 cm from the participant and pointed toward the face of the subject. The sample frequency was 10 Hz.

### 2.3. fNIRS Instrumentation

The fNIRS measurements were performed employing the Octamon fNIRS device (Artinis Medical Systems, Einsteinweg, The Netherlands). This device covered the pre-frontal cortex of the brain with 8 measurement channels resulting from 2 detectors and 8 bicolor Light Emitting Diodes (LED) at 760 and 850 nm of wavelengths. The sample frequency was 10 Hz.

### 2.4. IRT Tracking Procedure

The IRT tracking procedure was implemented for the purpose to follow the movement of the fNIRS detectors over time.

**Figure 1:**
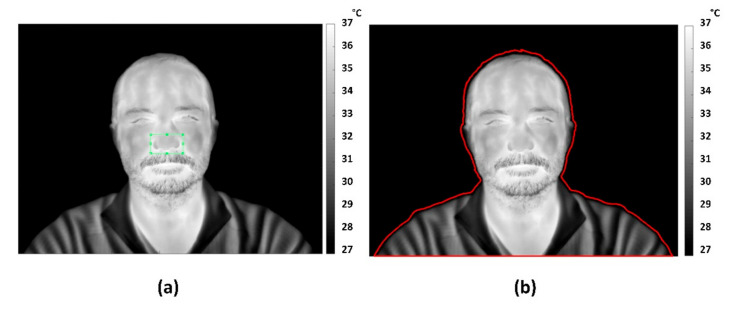
(**a**) Rectangular master ROI (mROI) initialized by the user on the first frame of the thermal video; (**b**) segmentation of thermal image through Otsu method.

The method is based on the evaluation of the 2-D cross-correlation between a target template (TT), chosen by the user in the initial frame of the thermal video, and the following frames. The whole interface and the code have been developed using the Matlab R2013a^®^ platform. The user is asked to select a rectangular ROI (master ROI-mROI) on the initialization frame (Figure 1a), in order to define the TT reference region to be tracked.

Each frame of the video is segmented by Otsu’s method [44] to separate the soft-tissue (i.e., the skin) from the background. This is done to ensure that the research for the best-matching ROI is computed only on a meaningful portion of the whole frame to speed up the computational process (Figure 1b).

For each frame, both target template and image under test (IUT) are decomposed through Gaussian pyramid decomposition [45]. Through this approach, the cross-correlation could be computed over a smaller size region with respect to the template size. In fact, Gaussian pyramid decomposition consists of a sequence of low-pass filters, whose order depends on the chosen level of decomposition, allowing the under-sampling of image pixel. The level of the Gaussian pyramid decomposition of the present tracker was 1, which allows us to reduce a 240 × 320 pixels image to a 120 × 160 pixels size.

To speed up the tracking process, the cross-correlation between the template and the target regions is calculated in the frequency domain and not in the spatial one, according to the procedure developed by Lyon et al. [46]. The location of the maximum value of the correlation coefficient frame by frame corresponds to the center location of the mROI across time.

In the initialization phase, the user can decide to draw one or more elliptic ROIs (slave ROIs, sROIs), that will follow the movement of the mROI, translating with it.

**Figure 2:**
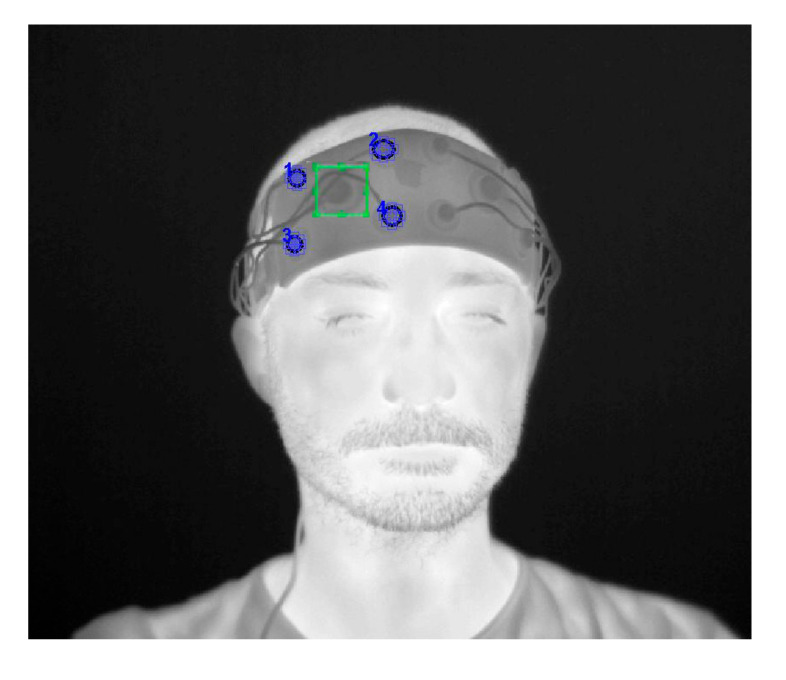
Initialization of one detector mROI and the sources sROIs.

For the purpose of this study, the mROI was placed over the detector and slave sROIs were placed on the sources of the fNIRS system, forming measurement channels (Figure 2). Since the developed software allows to define only one mROI, this operation is repeated for each detector.

**Figure 3:**
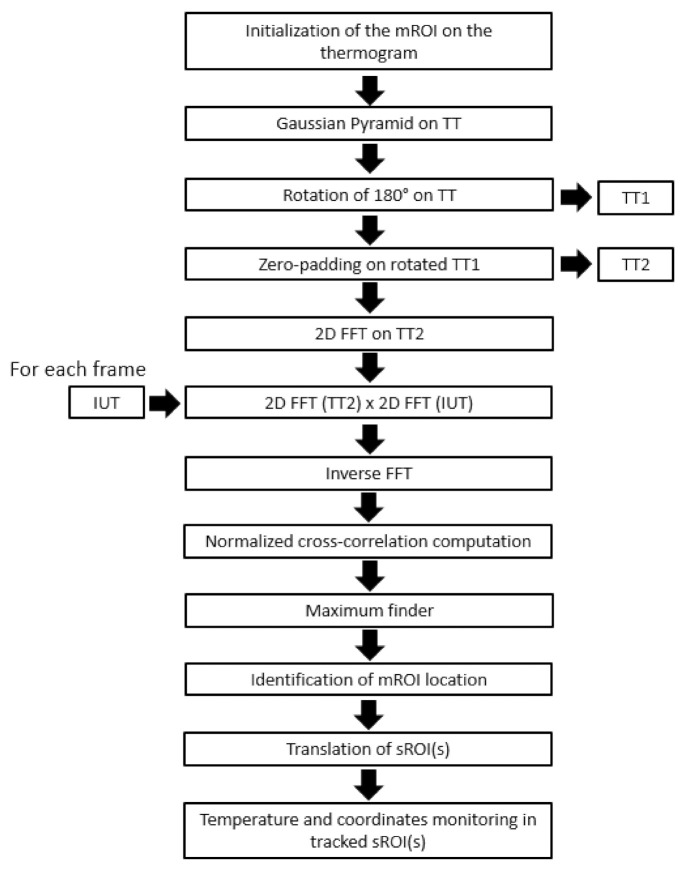
Flow chart for the tracking process.

The whole procedure for the tracking process is summarized in Figure 3.

The correlation coefficient between the initialized TT and the best region found over time provides the goodness index for the present tracking procedure. A threshold value for correlation coefficient is set before starting with the tracking algorithm (γ_th_). When the algorithm fails to find the tracked ROI with γ ≤ γ_th_ (γ_th_ = 0.995), the relative frames are discarded (a not a number—NaN—output is defined) and the algorithm re-starts to record the thermal signal and the ROIs position only when the correlation γ exceeds the threshold value. The correlation coefficient γ could decrease in case of out-of-the-plane rotational movement of the head or when there is a spatial occlusion. A specific threshold value of γ_th_ = 0.995 has been chosen on the basis of the performances of the IR tracker, which showed good reliability when the correlation is higher than this specific value. The specific threshold was set in a validation procedure, on thermal videos with spatial occlusions and rotational movement of the head. The overall best accuracy of the IR tracker was reached for the specific threshold reported (i.e., for γ_th_ ≥ 0.995).

The tracking procedure was developed to provide the temperature time course of sROIs. For the purpose of this study, the tracking procedure delivered as output the coordinates of the center of the optodes’ sROIs to be employed for the noise correction algorithm. Specifically, the x and y components of the motion of the center of the sROIs were combined to obtain the resultant motion. Of note, the algorithm did not fail to track the sROIs during the experiment, since the subjects were always in the camera’s field of view.

### 2.5. fNIRS Motion Artefacts Correction Algorithm

**Figure 4:**
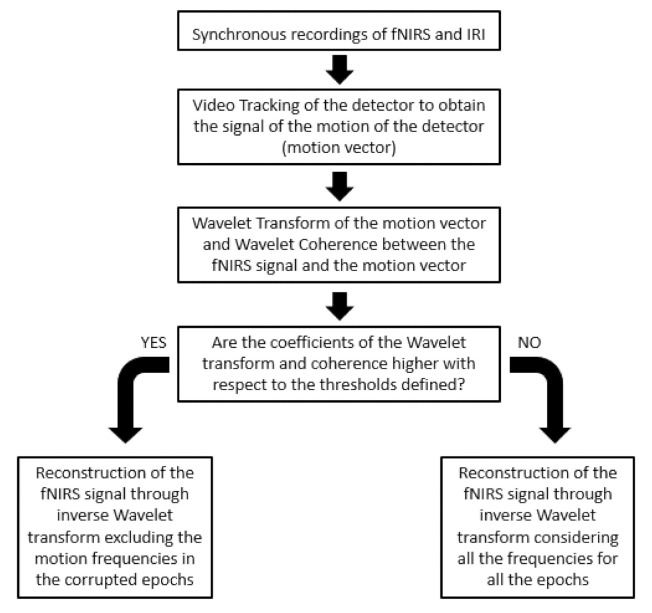
Flow chart of the motion correction algorithm.

The motion correction algorithm takes as input the signal of the motion of a fNIRS detector and the fNIRS signal (i.e., HbO_2_ and Hbb). The algorithm was developed in R2020a^®^ platform. The CWT is computed using the analytic Morse wavelet with the symmetry parameter equal to 3 and the time-bandwidth product equal to 60. The generalized Morse wavelets were preferred because of their time and frequency localization performance and their capability in isolating and extracting features in the time–frequency domain [47]. Moreover, CWT was preferred with respect to DWT because of its more fine-grained resolution. In fact, in order to identify the motion artifacts and their frequency content, a detailed time-frequency analysis and a precise localization of signal transients were necessary. The WCOH is computed using the analytic Morlet wavelet. In the algorithm, the CWT of the signal of the detector’s movement (named motion vector) and of the fNIRS signals was computed together with the WCOH between the motion vector and the fNIRS signal. A threshold of the coefficient of the CWT and WCOH were set in order to identify the frequency components that could be related to the head movements for each time point. Then, the functional signal was reconstructed by means of an inverse continuous wavelet transform (ICWF) excluding the frequency components associated to the motion artifacts. Figure 4 reports the algorithm developed for the motion correction.

**Figure 5:**
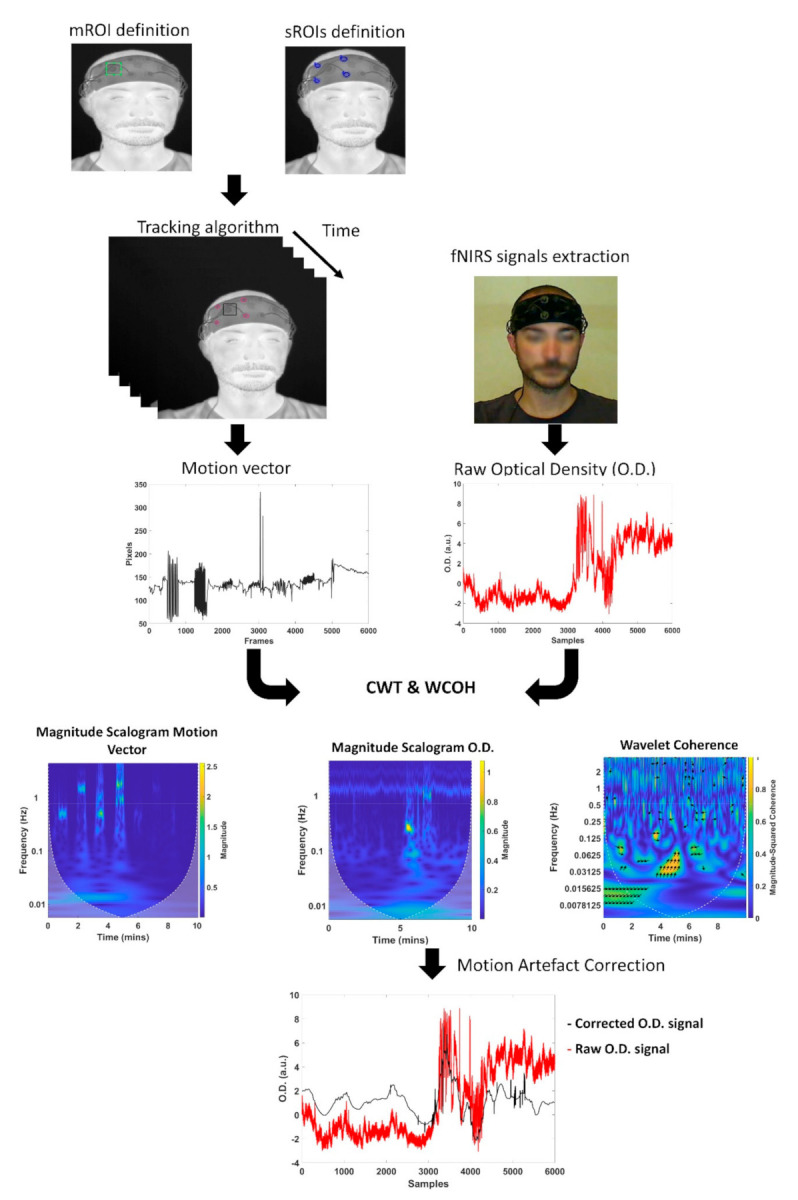
Procedure for data processing and motion artifacts correction.

The whole procedure developed for the motion artifacts correction is described in Figure 5.

### 2.6. Validation of the fNIRS Motion Artifacts Removal Algorithm

The participants were asked to perform some movements that are the most common source of artifacts for fNIRS signals (i.e., yawn, tilt of the head, frowning) in a random manner for 5 min. It was preferred to not perform a controlled movement of the head in order to replicate motion artifact as similar as possible to motion artifacts of the fNIRS signals. fNIRs and IRT were simultaneously collected.

A boxcar simulating a block paradigm was convolved with HRF to simulate the cortical activation in response to a block paradigm. The canonical GLM analysis used for fNIRS data analysis [48,49] was employed to test the performance of the proposed method and to compare its outcome with those of other motion correction algorithms (i.e., wavelet based [17], principal component analysis (PCA) based [14], spline based [16], and correlation based signal improvement (cbsi) method [50]). The mean squared error (MSE) between the HRF and the ODs, and an estimated signal to noise (SNR) were computed. Particularly, the SNR was evaluated by dividing the beta value (β) delivered by the GLM when the HRF was used as regressor, for the standard deviation of the OD during the resting periods (σ_rest_).

SNR = β/σ_rest_ (1)

MSEs and SNRs were computed for each channel, subject, and correction method. Moreover, the beta values and the associated t-scores obtained through the GLM analysis were compared with those obtained from the non-corrected data and the signal corrected using the reference procedures. This comparison allowed to test whether the proposed algorithm could improve the capability to assess the cortical activation.

Of note, these metrics were employed to investigate the optimal threshold for the CWT and WCOH to employ for the artifacts detection. Specifically, 5 thresholds (i.e., 0.4, 0.5, 0.6, 0.7, 0.8) were tested.

## 3. Results

### 3.1. IR Tracking Performances

The time consumed to track each frame of the IR video was evaluated over time, comparing the procedure considering or not considering the segmentation process. The time consumed by the segmentation process was not relevant compared to the procedure without segmentation. In fact, while the tracking procedure without Otsu’ segmentation required 0.054 s per frame, the procedure with the segmentation needed 0.073 s per frames to work. Thus, the difference in time was only 0.019 s/frames.

Regarding the comparison with other developed IR trackers, the present algorithm showed good performances, reporting a processing frequency rate of 18 Hz/14 Hz (with/without segmentation) on an Intel(R) Core(TM) i5 CPU computer with 8.00 GB RAM. Previously developed IR tracking algorithms reported lower processing frame rate of 1 Hz [40] or 6 Hz/12 Hz (single/multi-threading performance) of [41]. However, the performance of the present algorithm is lower than that declared in [43], in which the processing frame rate was higher than 25 Hz, but the system used was a PentiumIV 4-core computer, with 4 GB memory, that is more performing with respect to the system used for the present IR tracker.

### 3.2. Statistical Validation of the Motion Artifacts Removal Algorithm

**Figure 6:**
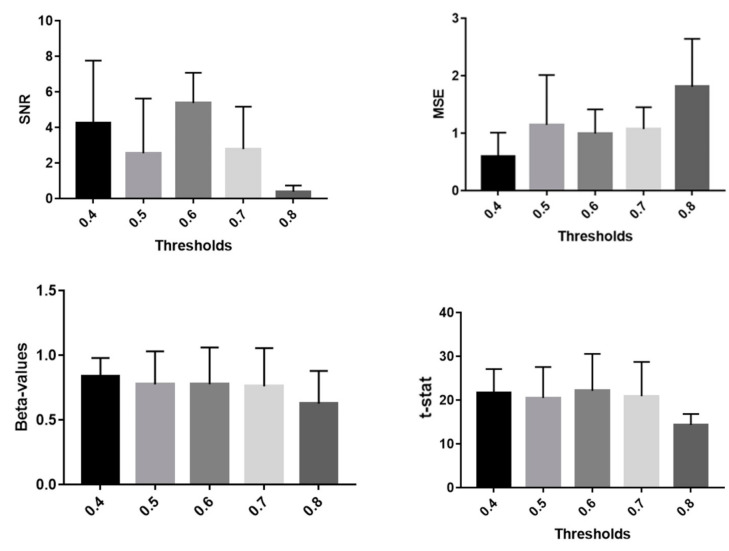
Average and standard deviation of the metrics employed to test the performances of the algorithm as a function of the chosen threshold.

Figure 6 reports the SNR and MSE (mean value and standard deviation) obtained with the proposed method testing 5 thresholds for the acceptance of the CWT and WCOH intensities. It was chosen to employ 0.6 as thresholds for further analysis.

**Figure 7:**
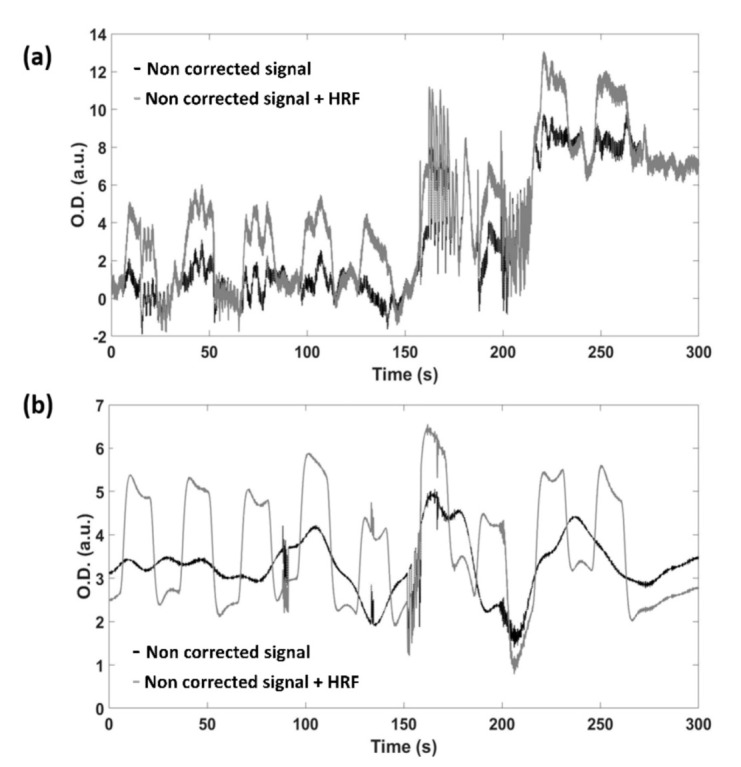
Example of one signal obtained by adding the synthesized HRF in the experimental paradigm simulated to the real resting state fNIRS data, before (**a**) and after (**b**) the application of the artifacts removal algorithm.

For further analysis, the threshold of 0.6 was employed since it shows the highest SNR. Figure 7 reports an example of the signals obtained by adding the synthesized HRF to the real resting state fNIRS data, before (Figure 7a) and after (Figure 7b) the application of the artifacts removal algorithm.

**Figure 8:**
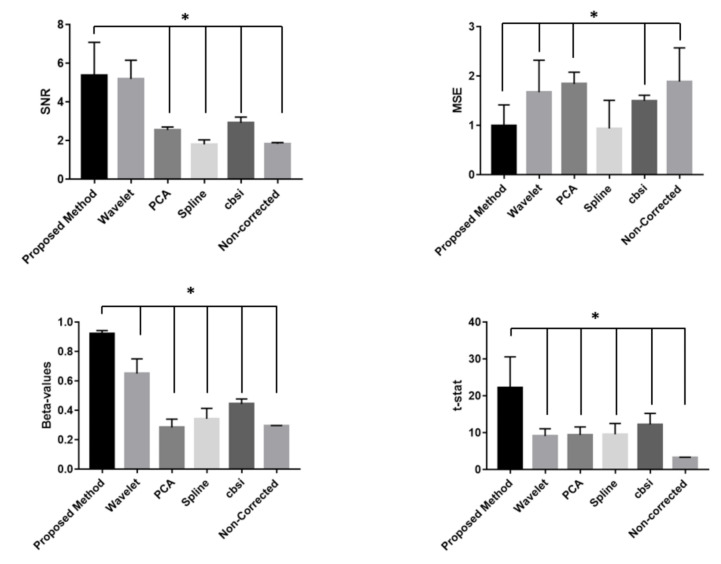
Average and standard deviation of the metrics employed to compare the motion artifacts correction. Statistically significant comparisons of paired *t*-tests are marked with an asterisk.

Figure 8 shows the MSE and SNR (average and standard deviation) obtained comparing the proposed method, the wavelet-based validated procedure and the non-corrected signal.

Table 1 reports the results of the paired *t*-test related to the metrics evaluated to investigate the method’s performance. The results are shown also in Figure 8 (in which statistically significant comparisons are marked with an asterisk).

## 4. Discussion

The aim of the present study was to validate a method for the motion artifacts correction of fNIRS signals. The method is based on a video tracking procedure developed for IRT recordings. The tracking procedure allowed us to estimate the motion of the optodes and to evaluate, by means of CWT and WCOH, its influence on the fNIRS signals. Five thresholds were tested to investigate the optimal value for the identification of the motion artifacts. Good results were obtained for all the thresholds investigated, but 0.4 and 0.6 showed the lowest MSE and highest SNR, respectively. The choice of employing 0.6 in this paper was dictated by the highest SNR obtained, but it is only indicative. In fact, it should be investigated whether the optimal threshold depends on the kind of motion to be corrected. Furthermore, the effect of defining different thresholds for the WCT and WCOH coefficients on the algorithm performance should be also deepened.

When comparing the performance of the proposed method with those of validated procedures, the metrics considered as indicative of the quality of the signals (i.e., SNR, MSE, beta values and t-stat) demonstrated statistically significant improvement of the data recorded. Of note, the SNR of the proposed method and that of the Wavelet-based procedure and the MSE obtained with the developed method and that of the spline-based procedure did not show statistically significant difference.

Multimodal fNIRS-IRT could provide information regarding the cortical activation and the psychophysiological state of the individual. In fact, it is known that some cortical regions and the autonomic nervous system are concurrently activated [51], especially during cognitive tests [52,53]. Furthermore, the IRT signal could be used also to assess and correct autonomic contaminations in the fNIRS signal [29,54,55,56]. In fact, it should be stressed that IRT recordings can provide information regarding the breathing rate [57,58] and the superficial perfusion [59] that could influence the fNIRS recordings.

Notably, the employment of IRT to track the optodes provides good performances also in poor lighting conditions. This feature could be fundamental for those system that do not have the correction for environmental light and to prevent the detectors saturation. However, it should be highlighted that with good lighting conditions, tracking procedures developed for visible videos could be used to this aim as well. The advantage of employing RGB cameras with respect to IRT relies on their low cost and on the availability of several validated tracking algorithms. However, the physiological information of the facial skin temperature is lost.

The motion artifacts correction algorithm proposed was validated employing real fNIRS data acquired at rest during the execution of random movements. By adding a fic-tious experimental paradigm to the collected fNIRS data (i.e., by adding HRFs convolved with a boxcar function simulating a block paradigm), it was possible to evaluate the improvements of the SNR and MSE related to the application of the algorithm. Moreover, a GLM based analysis was performed employing as regressor the simulated cortical activation, evaluating the beta-values and the associated t-stat. The results demonstrated an overall improvement of the quality of the signals after the administration of the algorithm, and, consequently, a better investigation of the cortical activation by means of the GLM analysis. It should be stressed that the proposed algorithm does not completely remove all the motion artifacts (Figure 7). Generally, motion correction algorithm trade off sensitivity with specificity, with algorithms heavily correcting for motion also affecting the underlying functional activity. However, the importance of the motion artifact correction is to deliver reliable statistical results of the cortical activation. The results demonstrated that the proposed method increases the capability of the GLM based approach to assess the cortical activation, demonstrating its effectiveness in improving the statistical analysis of the fNIRS signal. The developed method allows us to overcome some limitations of previous methods of motion artifacts correction based on the employment of an additional signal not sensitive to the cortical activation. Firstly, these approaches accept the assumption that the movement affect equally all the channels in a monotonic relation. However, some movements may corrupt only few channels, and the corruption could be not monotonically related to the movement. The developed method allows to investigate separately the movement of each detector and source, highly improving the removal of the motion artifacts. It should be highlighted that the proposed algorithm for the motion correction tracks the detectors’ movement (mROIs) and the movement of the sources (sROIs) is defined following the master ROI. Hence, the correction is limited to the detectors’ movement, but it should be possible that the sources move whereas the detectors are still. In this peculiar case, the proposed algorithm is ineffective. To independently assess the motion of the light sources, a master ROI should be defined for each source. It is worth to highlight that the developed tracking algorithm allows to define only 1 mROI at a time, thus tracking independently each source could be heavily time consuming. Indeed, further studies should be focused on the improvement of the tracking algorithm (e.g., implementing the possibility to define several master ROIs concurrently) in order to improve the motion artifact algorithm performance, taking into account also the sources’ movement. With such an improvement, it could be investigated if the residual motion artifacts observable on the signals after the correction are due to the uncoupled movement of sources and detectors.

Secondly, this typology of motion artifacts correction algorithms may not identify permanent shifts in light intensity after a movement. However, the results suggest that the method is able also to reduce the shift due to a permanent displacement of the optodes (Figure 7), but the experimental procedure proposed was not focused on this kind of artifacts, hence it is not possible to reliably estimate the method’s performance in this case. Further studies are needed on this topic.

One limitation of the proposed method relies on the difficulties to frame all the optodes. In the described application, the fNIRS system was composed of two detectors in the pre-frontal cortex, hence one IRT camera was sufficient to shoot all the helmet. However, for whole-head systems it could be necessary to use more than one camera and to provide a synchronization among them. In this situation, it could be desirable the employment of one IRT camera to shoot the face of the participant, in order to preserve the thermal physiological information, and other RGB cameras to frame the parietal and occipital optodes.

Another limitation is related to the employment of this method for outdoor applications, where it is not possible to shoot the subject’s face and to properly track the optodes. This limitation could be overcome thanks to the improvement of IRT technology that allowed to develop compact IRT cameras that could be installed in mobile phones [60,61,62], on robots [63], and in very small environments, such as cars [64,65].

Finally, it is worth highlighting that IRT cameras are indeed more expensive with respect to visible cameras. This issue could be solved enhancing the quality of videos acquired through low cost IRT cameras [66], thus improving the tracking algorithms’ performance when applied to low resolutions IRT videos.

## 5. Conclusions

In the fNIRS research field, it is well known that the subjects’ head movement can corrupt the fNIRS signals because of the decoupling between the optodes and the scalp, producing an abrupt modification of the light intensity. Besides improving the optodes–scalp coupling, the development of a motion artifacts correction algorithm is an essential point, although revealing quite a challenging issue. This paper reports about the development of a motion artifact correction algorithm for fNIRS signals based on wavelet transform, wavelet coherence and video tracking for IRT recordings. The motion artifact correction algorithm was validated on resting-state fNIRS signals with HRFs added simulating a block paradigm. The results demonstrated good performances of both the IR tracking and of the motion correction algorithm. It has been possible to improve the quality of the fNIRS signal and the detection of the cortical functional activation with very promising performances. This novel method can pave the way to multimodal fNIRS-IRT applications to concurrently detect the central and autonomic nervous system activity.

## Figures and Tables

**Table 1:** Paired *t*-test results (method proposed vs. non-corrected signal) associated to the metrics used to investigate the method’s performance, evaluated for the channels averaging all the subjects.

	Metric	*t*-Score	Degrees of Freedom	*p*-Value
Proposed Method vs. Non- corrected	SNR	5.766	7	6.87 × 10^−4^
	MSE	−9.352	7	3.32 × 10^−5^
	Beta-value	92.064	7	4.70 × 10^−12^
	t-stat	6.339	7	3.89 × 10^−4^
Proposed Method vs. Wavelet	SNR	0.249	7	0.811
	MSE	−8.768	7	5.05 × 10^−5^
	Beta-value	6.772	7	2.60 × 10^−4^
	t-stat	6.04	7	5.21 × 10^−4^
Proposed Method vs. PCA	SNR	5.986	7	5.50 × 10^−4^
	MSE	−4.827	7	0.002
	Beta-value	29.329	7	1.38 × 10^−8^
	t-stat	7.055	7	2.02 × 10^−4^
Proposed Method vs. Spline	SNR	5.444	7	9.62 × 10^−4^
	MSE	0.125	7	0.904
	Beta-value	22.537	7	8.57 × 10^−8^
	t-stat	6.937	7	2.23 × 10^−4^
Proposed Method vs. cbsi	SNR	4.571	7	0.003
	MSE	−6.445	7	3.51 × 10^−4^
	Beta-value	37.351	7	2.56 × 10^−9^
	t-stat	6.862	7	2.39 × 10^−4^

## Data Availability

The data presented in this study are available on request from the corresponding author. The data are not publicly available due to privacy issues.
